# Safety of Infliximab for the Eye Under Human T-Cell Leukemia Virus Type 1 Infectious Conditions *in vitro*

**DOI:** 10.3389/fmicb.2019.02148

**Published:** 2019-09-18

**Authors:** Minami Uchida, Koju Kamoi, Naoko Ando, Chenxi Wei, Hisako Karube, Kyoko Ohno-Matsui

**Affiliations:** Department of Ophthalmology and Visual Science, Graduate School of Medical and Dental Sciences, Tokyo Medical and Dental University, Tokyo, Japan

**Keywords:** human T-cell leukemia virus type 1, anti-TNF-alpha antibody, infliximab, ocular inflammation, uveitis

## Abstract

Use of biologics has been widely advocated for inflammatory diseases recently. Anti-tumor necrosis factor (TNF)-α antibody therapy is reportedly effective against ocular inflammation. However, side effects of TNF-α inhibition have been reported, particularly in the form of exacerbation of infections such as tuberculosis. Paradoxical reactions such as exacerbated inflammation are also well known. Around 20 million humans are infected with human T-cell leukemia virus type 1 (HTLV-1) globally, and this virus can cause adult T-cell leukemia, HTLV-1-associated myelopathy and HTLV-1 uveitis. As for ophthalmic concerns, it has not been identified whether anti-TNF-α antibody stimulates HTLV-1-infected cells and ocular cells to induce HTLV-1 uveitis in HTLV-1 carriers. Here we investigated the effects of anti-TNF-α antibody on ocular status under HTLV-1 infectious conditions using ocular cells and HTLV-1-infected cells *in vitro*. We used the ARPE-19 human retinal pigment epithelial cell line as ocular cells considered to play an important role in the blood-ocular barrier, and the MT2 HTLV-1-infected cell line. Jurkat cells were used as controls. Infliximab (IFX) was used as an anti-TNF-α antibody to achieve TNF-α inhibition. We evaluated the production of inflammatory cytokines and intercellular adhesion molecule (ICAM)-1, proliferation of ARPE-19, expression of TNF-α receptor (TNF-R) and HTLV-1 proviral DNA, and the percentage of apoptotic ARPE-19. Inflammatory cytokines such as interleukin (IL)-6, IL-8, TNF, and ICAM-1 were significantly elevated through contact between ARPE-19 and MT2. Treatment with IFX tented to inhibit TNF production, although the level of production was low, but changes in IL-6, IL-8, and ICAM-1 remained unaffected. Expression of TNFR was unaltered by IFX treatment. HTLV-1 proviral DNA was not significantly changed with treatment. No change in cell growth rate or apoptotic rate of ARPE-19 was seen with the addition of IFX. In conclusion, IFX did not exacerbate production of inflammatory cytokines, and did not affect expression of TNFR, proliferation of ARPE-19, HTLV-1 proviral load, or apoptosis of ARPE-19. These results suggest that IFX does not exacerbate HTLV-1-related inflammation in the eye and represents an acceptable treatment option under HTLV-1 infectious conditions.

## Introduction

Human T-cell leukemia virus type 1 (HTLV-1) is a retrovirus that infects an estimated 20 million people globally (Watanabe, 2011). Various HTLV-1 endemic areas exist around the world, and about 1 million HTLV-1-infected individuals live in Japan, mainly in southwestern regions such as Kyushu and the Okinawa islands (Satake et al., 2012). HTLV-1 causes adult T-cell lymphoma (ATL) (Uchiyama et al., 1977), HTLV-1-associated myelopathy (HAM) (Osame et al., 1986), and HTLV-1 uveitis (HU) (Mochizuki et al., 1992). HU is considered by ophthalmologists as one of the most common ocular inflammatory diseases in HTLV-1 endemic areas (Terada et al., 2017a). This pathological entity was established after a series of seroepidemiological, clinical, molecular biological, and virological studies (Kamoi and Mochizuki, 2012a,b). HU occurs with a sudden onset of floaters and foggy vision, and is classified as an intermediate uveitis (Takahashi et al., 2000). HU is considered to be caused by inflammatory cytokines produced by HTLV-1-infected T-cells accumulating in the eyes (Kamoi and Mochizuki, 2012b).

Biologics have been widely advocated for use against inflammatory diseases recently. In particular, anti-tumor necrosis factor (TNF)-α antibody therapy has seen wide use for rheumatoid arthritis (RA) (Bathon et al., 2000), psoriasis (Leonardi et al., 2003), ankylosing spondylitis (Gorman et al., 2002), and inflammatory bowel disease (Rutgeerts et al., 2005). In addition, anti-TNF-α antibody therapy has been reported as effective for ocular inflammation (Joseph et al., 2003). In the ophthalmological field in Japan, infliximab (IFX) has been approved for use in the treatment of Behçet disease since 2008, and adalimumab has been approved for use against non-infectious uveitis since 2016 (Diaz-Llopis et al., 2008). Anti-TNF-α antibody acts to neutralize activities against soluble TNF-α, block TNF-α binding to TNF receptors on cells, and induce apoptosis and arrest of the cell cycle in G0/G1 in TNF-α-producing cells (Lim et al., 2018).

Although significant efficacy of anti-TNF has been reported, side effects of TNF-α inhibition such as infection (Lim et al., 2018), malignant tumor (Wang et al., 2016), autoimmune disease (Perez-De-Lis et al., 2017), and demyelinating disease (Gill et al., 2017) have been reported. In particular, exacerbation of infection was a notable side effect, and a meta-analysis indicated that caution is needed for patients infected with tuberculosis, syphilis, toxoplasma, herpes, cytomegalovirus, bacteria, or fungi (Minozzi et al., 2016). In addition, paradoxical reactions such as exacerbation of inflammation are well known. Such paradoxical effects have mainly been reported as skin reactions, granulomatous diseases, and ocular inflammation (uveitis) (Wendling and Prati, 2014; Mylonas and Conrad, 2018).

However, problematically, the risks of administering TNF-α inhibitors to patients infected with HTLV-1 have yet to be clarified, because few basic and clinical studies have been conducted for this retrovirus. Clinically, cases of rheumatoid arthritis complicated with HTLV-1 infection reportedly did not show any sign of exacerbated infection, such as increased proviral load (PVL), after providing anti-TNF-α antibody therapy (Umekita et al., 2015). Basically, TNF-α inhibition showed no effect on a HTLV-1-infected cell line from patients with HAM *in vitro* (Fukui et al., 2017).

As mentioned above, several reports have examined the safety of anti-TNF-α antibody therapy in internal medicine fields, but no report from the ophthalmological field has clarified whether TNF-α inhibition can affect ocular conditions in HTLV-1 carriers. HU is one of the most important clinical entities among HTLV-1-infected individuals. As a result, the risk of induction of HTLV-1-related ocular inflammation (i.e., HU) following administration of anti-TNF-α antibody into HTLV-1 carriers is a concern warranting investigation. In addition, guidelines for the use of anti-TNF-α antibody have not mentioned screening for HTLV-1 infection prior to initiation of treatment.

The present study investigated the effects of anti-TNF-α antibody on ocular status among HTLV-1 carriers using an ocular cell line and an HTLV-1-infected cell line *in vitro*. The present investigation chose a retinal pigment epithelium (RPE) cell line as a representative ocular cell line, as the RPE plays a major role in the blood-ocular barrier and the maintenance of immunological homeostasis in the eye (Holtkamp et al., 2001).

## Materials and Methods

### Cell Lines, Cell Culture, and Human T-Cell Leukemia Virus Type 1 Infection *in vitro*

We used the ARPE-19 human retinal pigment epithelial cell line (American Type Culture Collection, Manassas, VA) as ocular cells, and the MT-2 cell line as an HTLV-1-infected T-cell line. We used Jurkat cells as a control T-cell line. MT2 and Jurkat cells were cultured in RPMI 1640 (Wako Pure Chemical Corporation, Osaka, Japan) supplemented with 10% fetal bovine serum (FBS) (GE Healthcare Japan, Tokyo, Japan) and 1% penicillin/streptomycin. ARPE-19 cells were cultured in minimum essential medium (Wako Pure Chemical Corporation) with the same supplements. All cell lines were incubated in a humidified incubator at 37°C under an atmosphere of 5% CO_2_. *In vitro* infection by HTLV-1 was performed using the standard co-culture method (Akagi et al., 1986; Graziano et al., 1987; Liu et al., 2006). Briefly, ARPE-19 cells were plated and co-cultured with three times the number of MT2 or Jurkat cells at 48 h using cell culture inserts (Thermo Fisher Scientific, Waltham, MA). 1.5 × 10^5^ ARPE-19 cells were used in cytometric bead assay (CBA) and annexin V assay. 2 × 10^4^ ARPE-19 cells were used in cell counts, TNF receptor analysis, and the measurement of HTLV-1 proviral load.

### Anti-Tumor Necrosis Factor-α Inhibitor

IFX (Mitsubishi Tanabe Pharma, Osaka, Japan) was used as an anti-TNF-α antibody, with 10 μg/ml/well, in line with a previously established *in vitro* method for HTLV-1-associated myelopathy experiments (Fukui et al., 2017).

### Cytometric Bead Assay

Cultured supernatants were examined using CBA human inflammation cytokine kits (BD Biosciences, San Jose, CA). Results were analyzed with FCAP Array version 3.0 software (BD Biosciences) according to the instructions from the manufacturer. Cytokines measured by the kits included interleukin (IL)-6, IL-8, IL-1β, IL-12p70, IL-10, and TNF.

### Cytokine Enzyme-Linked Immunosorbent Assays

To measure levels of soluble intercellular adhesion molecule (ICAM)-1 in supernatants, ELISA kits (R&D Systems, Minneapolis, MN) were used in accordance with the instructions from the manufacturer.

### Cell Counts

ARPE-19 cells (2 × 10^4^) were co-cultured with three times the number of MT2 or Jurkat cells with or without IFX. After 0, 24, 48, or 72 h of co-culture, we removed the supernatants, trypsinized ARPE-19, and counted the number of ARPE-19 cells under light microscopy.

### Anti-Tumor Necrosis Factor-α Receptor Analysis

Fluorescence-activated cell sorting (FACS) analysis was performed to examine the cell surface expression of TNF-R1 and TNF-R2, using fluorescein isothiocyanate (FITC)-conjugated anti-CD120a (TNF-R1) and anti-CD120b (TNF-R2) human monoclonal antibodies (MBL International, Woburn, MA). ARPE-19 cells were washed and trypsinized, then incubated with TNF-R1 and TNF-R2 antibodies according to the instructions from the manufacturer. Analysis was performed using a FACSCalibur flow cytometer and CellQuest software (BD Biosciences).

### Immunohistochemistry

ARPE-19 cells were cultured on glass bottom plates (AGC Techno Glass, Shizuoka, Japan) for 24 h, then co-cultured with MT2 and Jurkat cells using cell culture inserts (Thermo Fisher Scientific) for 48 h. After three washes with phosphate-buffered saline (PBS) (Wako Pure Chemical Corporation), ARPE-19 cells were fixed by cold fixing buffer (methanol/acetone, 1:1) at −20°C for 20 min and blocked with 10% FBS in PBS for 15 min. Cells were then incubated in the diluted primary antibodies for 1 h at room temperature, followed by incubation with Alexa fluor488-labeled anti-rabbit secondary antibody (Abcam, Tokyo, Japan) along with 4′,6-diamidino-2-phenylindole dihydrochloride (Cosmo Bio, Tokyo, Japan) incubation for 1 h at room temperature. The following antibodies were used as primary antibodies: TNF receptor 1 polyclonal antibody (Bioss Antibodies, Woburn, MA) and TNF receptor 2 polyclonal antibody (Proteintech, Chicago, IL). We scanned using a TCS-SP8 microscope (Leica Micro Systems, Wetzlar, Germany).

### Measurement of Human T-Cell Leukemia Virus Type 1 Proviral Load

DNA was prepared from each sample using EZ1 Virus Mini Kits v2.0 (Qiagen, Hilden, Germany) according to the instructions from the manufacturer. Quantitative real-time polymerase chain reaction (PCR) assay was used to measure the PVL of HTLV-1 in cells, as described previously (Matsuda et al., 2005; Fukui et al., 2017). PVL was quantified using the HTLV-1 Tax primer (forward, 5’-CCCACTTCC CAGGGTTTGGA-3′; reverse, 5’-GGCCAGTAGGGCG TGA-3′) and probe (5’-FAM-CCAGTCTACGTGTTTGGA GACTGTGTACA-TAMRA-3′). Glyceraldehyde-3-phosphate dehydrogenase was used as the internal control.

### Annexin V Staining

FACS analysis to evaluate of apoptosis and cell death using Annexin V-FITC was performed using assay kits (MBL, Nagoya, Japan) according to the instructions from the manufacturer. Apoptotic cells were defined by detecting propidium iodide-negative, annexin V-positive cells. The rate of apoptotic cells among all cells was calculated by FACSCalibur flow cytometer and CellQuest software (BD Biosciences).

### Statistical Analysis

Student’s unpaired *t*-test or Welch’s unpaired *t*-test after Bonferroni correction for multiple testing, which adjusts the values of *p* by multiplying them by the number of tests (Krzywinsk and Altman, 2014), was used to determine significant differences in the levels of cytokines, number of ARPE-19 cells, and percentage of apoptotic cells. Values of *p* < 0.05 were considered significant. All statistical analyses were performed using EZR (Saitama Medical Center, Jichi Medical University, Saitama, Japan). More precisely, EZR is a modified version of R Commander (version 1.6-3) designed to add statistical functions used frequently in biostatistics.

## Results

### Inflammatory Cytokines and Intercellular Adhesion Molecule-1

**Figure 1:**
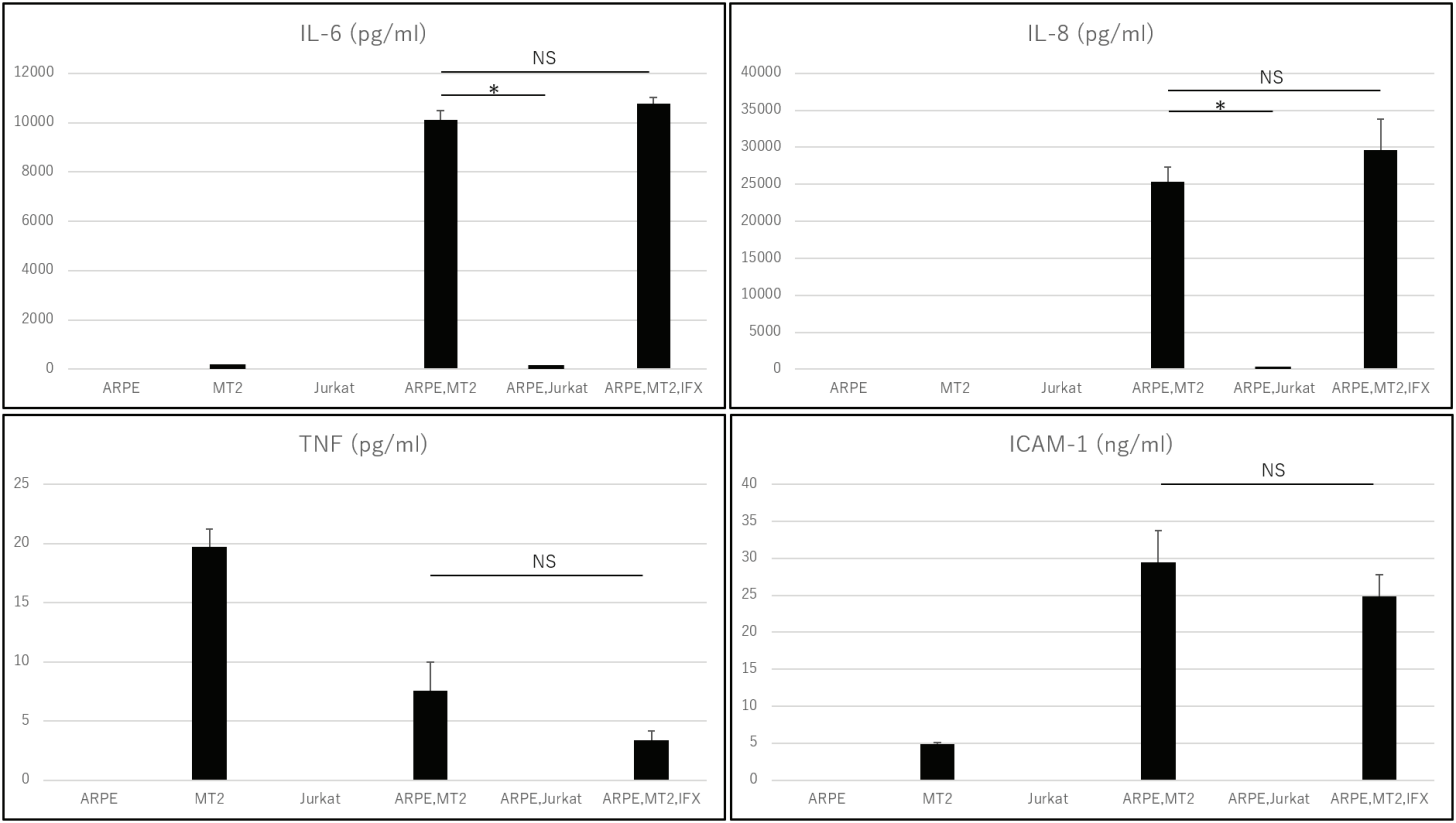
Inflammatory cytokines and ICAM-1 secreted by ARPE, MT2, Jurkat, and ARPE-19 co-cultured with MT2 or Jurkat, and ARPE-19 co-cultured with MT2 with addition of IFX at 48 h. Although TNF tends to be inhibited by IFX, no significant difference is detected (*p* = 0.14). Data were obtained from three independent biological replicates performed in triplicate. Error bars represent standard deviations (*p < 0.05; NS, not significant).

Levels of IL-6, IL-8, IL-1β, IL-12p70, IL-10, TNF, and ICAM-1 secreted by each of ARPE-19, MT2, Jurkat, ARPE-19 co-cultured with MT2, and ARPE-19 co-cultured with Jurkat were measured (Figure 1). MT2 spontaneously secreted IL-6, TNF, and ICAM-1. Levels of IL-6, IL-8, TNF, and ICAM-1 in the ARPE-19 co-cultured with MT2 were increased significantly more than those co-cultured with Jurkat. Compared to MT2 alone, levels of IL-6, IL-8, and ICAM-1 were increased. On the other hand, levels of TNF decreased in the ARPE-19 co-cultured with MT2. Levels of IL-12p70, IL-1β, and IL-10 were below the limits of detection under all conditions.

### Changes in Inflammatory Cytokines and Intercellular Adhesion Molecule-1 on ARPE-19 Treated With Infliximab

Changes in the levels of inflammatory cytokines and ICAM-1 secreted by ARPE-19 co-cultured with MT2 under treatment with IFX were examined. IL-6, IL-8, and ICAM-1 were unaffected by addition of IFX. TNF tended to be inhibited, but did not reach the level of statistical significance for suppression (*p* = 0.14). Levels of IL-12p70, IL-1β, and IL-10 were below measurable limits under all conditions (Figure 1).

### Changes in Cell Growth Rate in ARPE-19 Treated With Infliximab

**Figure 2:**
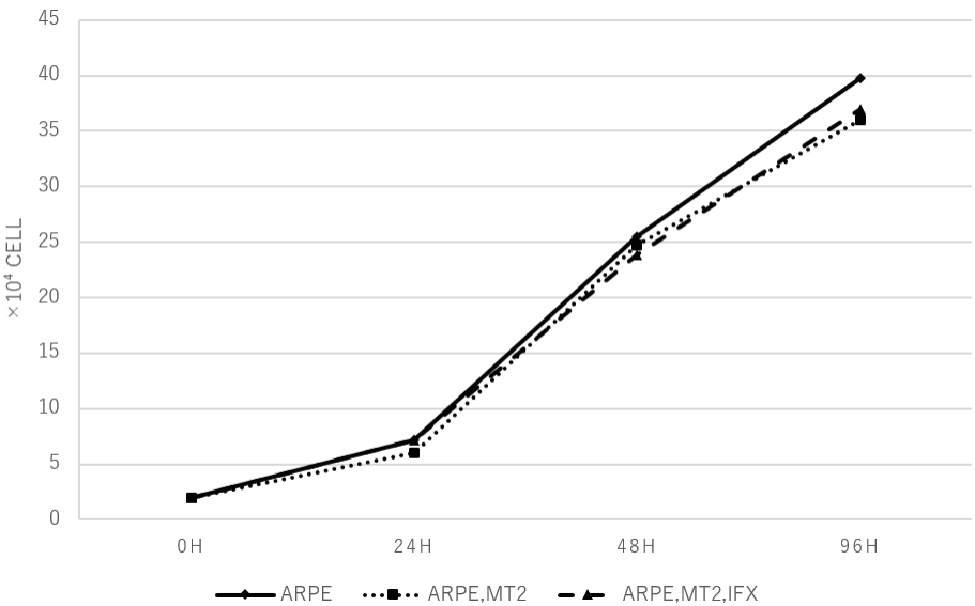
The number of ARPE-19 cells per well at 0, 24, 48, and 96 h. ARPE-19 cells increase in a time-dependent manner and no significant differences among the number of ARPE-19 cells co-cultured with MT2 cells with or without IFX are seen at each point. We conducted independent experiments and plotted the average values.

Changes in the cell growth rate of ARPE-19 co-cultured with MT2 treated with IFX were examined. Numbers of ARPE-19 cells after co-culture for 0, 24, 48, and 96 h increased in a time-dependent manner, and counts of ARPE-19 co-cultured with MT2 also increased in a similar manner. ARPE-19 cells co-cultured with MT2 tended to be slightly less numerous than ARPE-19 cells alone after co-culture for 96 h, but no significant differences were found. Furthermore, no significant differences were apparent between the numbers of ARPE-19 cells co-cultured with MT2 treated with and without IFX (Figure 2).

### Expression of TNF-R1 and TNF-R2 on ARPE-19 Treated With Infliximab

**Figure 3:**
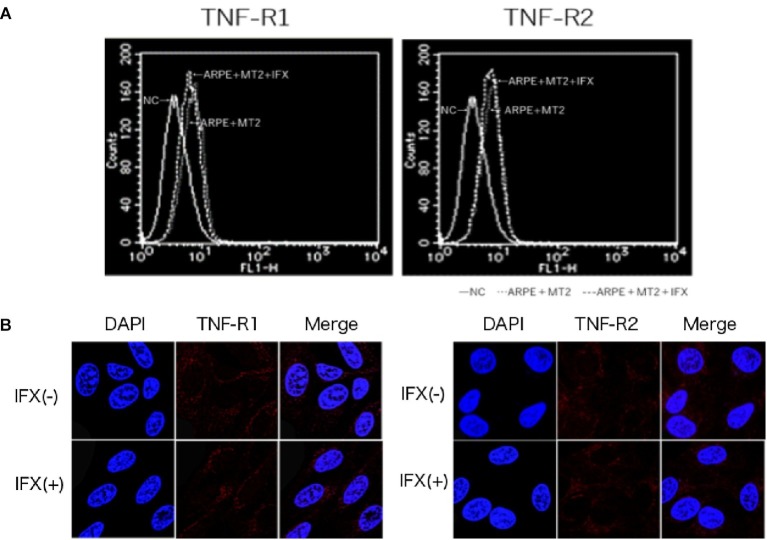
**(A)** Histogram of TNF-R1 and TNF-R2 on ARPE-19 in FACS analysis. Histograms of TNF-R on ARPE-19 and ARPE-19 after co-culture with MT2 with or without IFX. Results are representative of four experiments. **(B)** Expression of TNF-R1 and TNF-R2 on ARPE-19 in immunohistochemical staining. No significant changes in expression of TNF-R1 or TNF-R2 are seen for ARPE-19 treated with or without IFX.

To identify changes in the expression of TNF-R1 and TNF-R2 caused by IFX, we performed FACS analysis and immunohistochemical staining on ARPE-19 co-cultured with MT2 with addition of IFX. In FACS analysis, the number of TNF-R1 and TNF-R2 on ARPE-19 cells was unchanged by co-culture with MT2 or Jurkat cells (data not shown). Likewise, there were no differences in the histograms of TNF-R1 and TNF-R2 on ARPE-19 by treatment with IFX (Figure 3A). Furthermore, in immunohistochemical staining, expression of TNF-R was unaltered by IFX treatment (Figure 3B).

### Detection of Human T-Cell Leukemia Virus Type 1 Proviral DNA in Human T-Cell Leukemia Virus Type 1-Infected ARPE-19 Cells Treated With Infliximab

**Figure 4:**
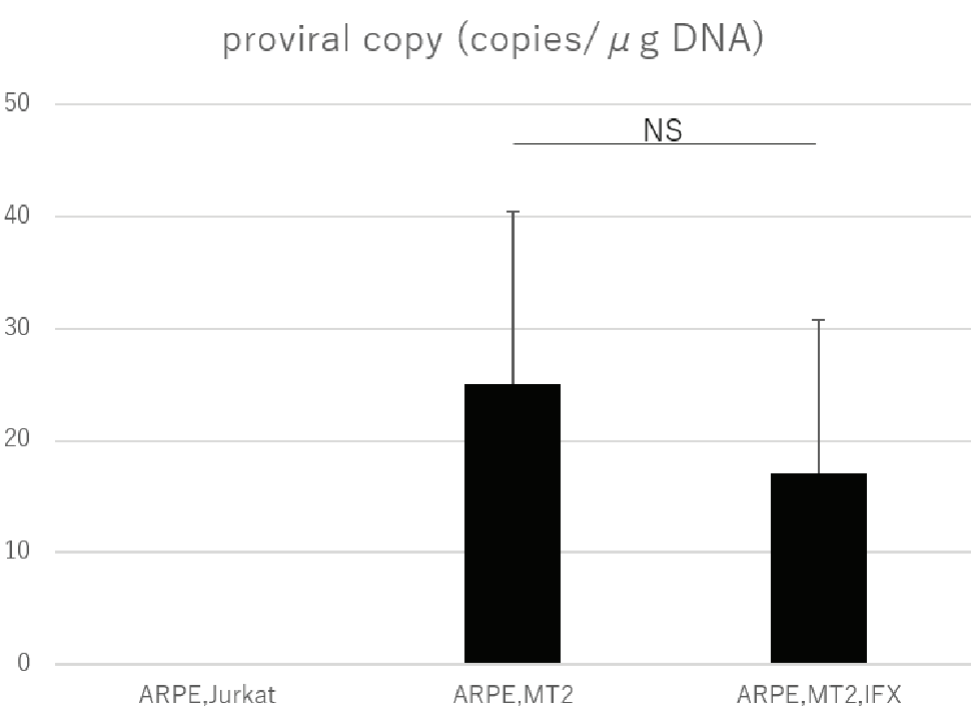
PVL in ARPE-19 cells co-cultured with Jurkat, MT2, or MT2 treated with IFX. The PVL of HTLV-1-infected ARPE-19 is not increased, but is slightly decreased by IFX. Error bars represent standard deviations (NS, not significant).

PVL was measured by real-time PCR to assess whether IFX affected HTLV-1 related disease progression in ARPE-19 cells. PVL in ARPE-19 cells co-cultured with MT2 cells was significantly increased compared to that in co-culture with Jurkat cells. As for controls, undetectable PVL was confirmed in ARPE-19 cells co-cultured with Jurkat cells. In HTLV-1-infected ARPE-19, PVL did not increase with IFX treatment (Figure 4).

### Assessment of Apoptosis in Human T-Cell Leukemia Virus Type 1-Infected ARPE-19 Cells Treated With Infliximab

**Figure 5:**
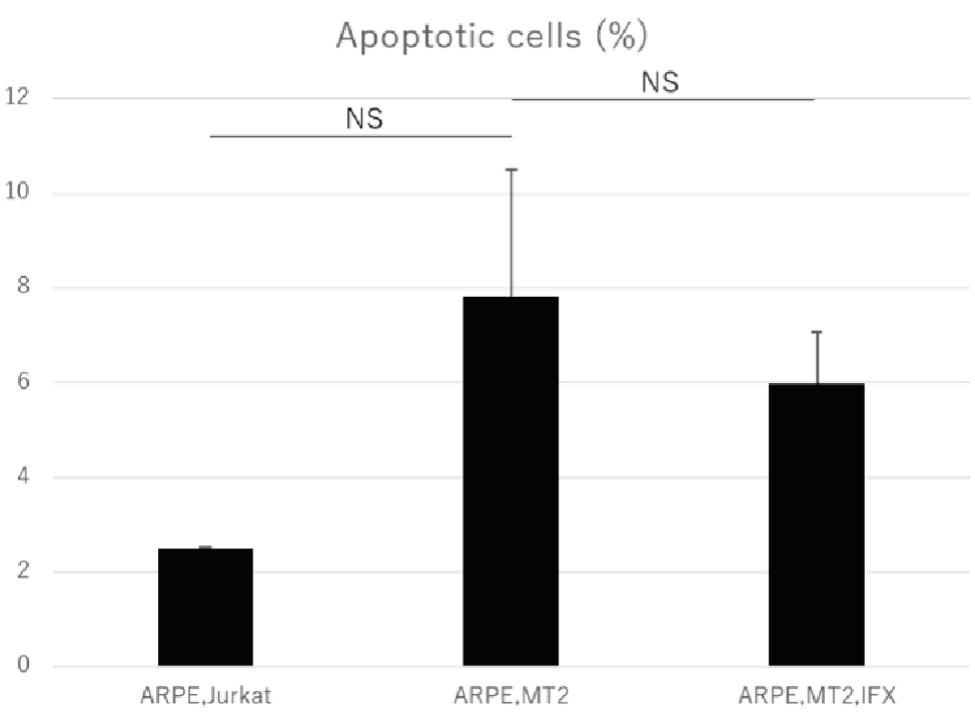
Rate of apoptotic ARPE-19 cells in co-culture with Jurkat, MT2, and MT2 treated with IFX. No significant change in apoptotic rate of ARPE-19 is seen with IFX; rather, there is a slight decrease with IFX. Error bars represent standard deviations (NS, not significant).

Annexin V assay was performed to assess the apoptosis in ARPE-19 co-cultured with MT2 and the effect of IFX on ARPE-19 was examined. The apoptotic rate of ARPE-19 co-cultured with MT2 tended to be increased compared to that in co-culture with Jurkat, but the difference was not significant (*p* = 0.10). No change in the apoptotic rate of ARPE-19 co-cultured with MT2 was seen with the addition of IFX (Figure 5).

## Discussion

Biologics are reportedly effective for ocular inflammation and have been used worldwide. Although HTLV-1 carriers are common all over the world, no basic studies have been conducted to clarify the safety of biologics for HTLV-1-infected patients in the ophthalmological field. In Japan, several decades have passed since the first biologic was applied to inflammatory diseases, and Japan is now one of the countries with the greatest use of biologics by physicians. A case has already been reported in which an RA patient with HTLV-1 infection experienced exacerbation of HTLV-1-associated conditions such as myopathy and uveitis after using a biologic agent (Terada et al., 2017b). Clarification of the safety of biologics on HTLV-1-infected patients is thus considered an urgent issue. This study focused on IFX, as the first biologic agent applied to inflammatory diseases, and clarified the effect of IFX on ocular conditions under an HTLV-1-infected status.

The intraocular concentrations of cytokines and chemokines greatly influence the pathophysiology of ocular inflammation (Mochizuki et al., 2013). The RPE plays an important role in the ocular-blood barrier (Holtkamp et al., 2001) and in the intraocular immune response by producing various inflammatory cytokines and expressing adhesion molecules such as ICAM-1 (Elner et al., 1992). Immunosuppressive and immunomodulatory therapies such as biologics have been reported to provide beneficial effects in patients with this disorder. IFX is an effective agent for various inflammatory conditions and many reports have indicated that IFX has potential for decreasing various cytokines in patients with inflammatory diseases. However, whether TNF-α inhibition can affect the eye of HTLV-1 carriers has not been clarified. In fact, paradoxical reactions, i.e., exacerbated inflammation, are also well known with anti-TNF-α treatment. In particular, ocular inflammation (uveitis) has been reported as a major paradoxical effect from treatment with anti-TNF-α antibody (Wendling and Prati, 2014). We therefore first checked the effect of IFX on inflammatory cytokines such as IL-6, IL-8, IL-1β, IL-12p70, IL-10, and TNF. Inflammatory cytokine investigations showed that levels of IL-6 and IL-8 increased when HTLV-1-infected T-cells came into contact with RPE (Figure 1). Those inflammatory cytokines resulted in inflammatory conditions in the eye, suggesting that HTLV-1 carriers potentially face a credible threat of intraocular inflammation. In such a state, however, IFX treatments did not affect levels of IL-6 or IL-8 (Figure 1). This means that IFX did not encourage acceleration of the inflammatory conditions that result in HU in the eyes of HTLV-1-infected patients.

As for TNF-α, our experiments showed that levels of this cytokine were low, but decreased further when HTLV-1-infected T-cells came into contact with RPE, compared to HTLV-1-infected T-cells alone (Figure 1). This phenomenon is related to the nature of RPE, which plays a role in local immunosuppressive ability to protect the blood-ocular barrier (Mochizuki et al., 2013). The result showed that suppression of TNF-α seems to be one of the characteristic features of the RPE response to HTLV-1 infection in a local immunosuppressive role. Although no significant reduction was identified, IFX treatment led to a relative reduction in TNF-α under this condition (Figure 1), providing positive evidence for IFX-induced suppression of ocular inflammation. However, this experiment was not specifically aimed at identifying the effectiveness of anti-TNF-α antibody for HTLV-1 uveitis, but rather at confirming the safety of anti-TNF-α antibody in terms of the ocular circumstances of HTLV-1 carriers.

We next focused on another important molecule, ICAM-1. ICAM-1 acts to mediate cell-to-cell interactions, (Lebedeva et al., 2005) and ICAM-1 has been reported as necessary for HTLV-1 infection. ICAM-1 has also been reported to be upregulated by HTLV-1 infection (Liu et al., 2006) and contributes to this efficient transmission (Barnard et al., 2005). From the viewpoint of ocular circumstance, it has been well known that increase of soluble ICAM is detected in the ocular fluid samples from patients with various types of uveitis, including Behçet disease-related uveitis which is a major targeted disease of infliximab. Our result showed that soluble ICAM-1 secreted by ARPE-19 was increased by contact with HTLV-1-infected cells, as expected (Figure 1). However, IFX treatment did not influence the level of soluble ICAM-1 under this condition (Figure 1), and was therefore estimated to play no role in further accelerating HTLV-1 infection or upregulation, which is consistent with results from an *in vitro* investigation of HTLV-1-associated myelopathy (Fukui et al., 2017).

Inhibition of IFX is mediated *via* interference of TNF binding to two known receptors, namely TNF-R1, which binds to soluble TNF, and TNF-R2, which binds to membrane-bound TNF. As shown in previous studies, TNF-R1 and TNF-R2 are expressed on the RPE (Kociok et al., 1998; Lee et al., 2015), and changes in these receptors are therefore thought to influence the function of the blood-ocular barrier through TNF-mediated inflammation. In the present study, FACS analysis and immunohistochemical staining identified no change in TNF-R1 or TNF-R2 expression on ARPE-19 by IFX treatment (Figures 3A,B). This suggests that IFX did not accelerate TNF-mediated alterations in the eye.

The PVL in peripheral blood is related to the development of HTLV-1-associated diseases (Demontis et al., 2013), and PVL is used as a marker for HTLV-1 disease progression (Moens et al., 2009). Many investigations have looked into PVL in peripheral blood, but information on PVL in human residential tissue and cells is scant. The present study therefore tried to check whether changes to PVL in RPE would occur under IFX administration. A previous investigation identified RPE as a potential reservoir for HTLV-1, as HTLV-1 proviral DNA was detected in ARPE-19 cells co-cultured with MT2 cells (Liu et al., 2006). Our study confirmed that proviral DNA in RPE was detectable in the co-culture experiment, as previously reported. More importantly, we were able to demonstrate that PVL in RPE was not increased by IFX treatment (Figure 4), indicating that IFX did not affect PVL in RPE. This result suggested that IFX was unrelated to HTLV-1 provirus-related disturbance, potentially leading to a breakdown of the blood-ocular barrier, in RPE.

An increase in apoptotic ARPE cells represents critical damage to the maintenance of immunological homeostasis in the eye (Mochizuki et al., 2013). In the present study, the apoptotic rate of ARPE-19 cells co-cultured with MT2 cells tended to be increased compared to that in co-culture with Jurkat cells, but no significant difference was identified (Figure 5). From one perspective, apoptosis contributes to suppression of cell carcinogenesis and control of infection spread (Tabakin-Fix et al., 2006), so this apoptotic rate in the case of ARPE-19 co-cultured with MT2 might be considered as an infection-control reaction of ARPE. This result also may be related to the slightly reduced proliferation of ARPE-19 co-cultured with MT2 at 96 h (Figure 2). In addition, the apoptotic rate of HTLV-1-infected ARPE-19 cells was not increased by the addition of IFX (Figure 5). This result indicated that further damage did not occur in HTLV-1-infected ARPE-19 cells with IFX treatment.

Some limitations to this study merit attention when considering the potential effects of IFX therapy in patients with ocular inflammation complicated by HTLV-1 infection. For instance, in our experiments involving inflammatory cytokines, we did not observe the increases in IL-1 and IL-10 in co-cultures of ARPE-19 and MT2 that were described in previous clinical reports (Sagawa et al., 1995). This *in vitro* investigation using cell lines might not accurately reflect all aspects of clinical events. As we placed particular focus on changes to ocular tissue in this investigation, we did not examine the risk of ATL induction using IFX. Although previous basic and clinical papers have not indicated any increased PVL in peripheral blood mononuclear cells with IFX treatment (Umekita et al., 2015; Fukui et al., 2017), we would next focus on the alteration of HTLV-1-infected T cells by IFX treatment through Tax analysis, DNA damage, and cell-cycle progression. In conjunction with basic research, clinical long-term tracking investigations of IFX-treated patients might also be needed.

## Conclusion

IFX did not exacerbate the production of inflammatory cytokines, and did not affect the expression of TNFR, cell proliferation, HTLV-1 PVL, or apoptosis on RPE. These results suggest that IFX did not exacerbate HTLV-1-related inflammation in the eye and represents an acceptable treatment option, at least according to HTLV-1 infectious conditions *in vitro*.

## Author Contributions

MU performed the experiments and wrote the draft of the manuscript. KK designed the experiments, analyzed the data, and wrote the manuscript. NA, CW, and HK performed the experiments. KO-M contributed to analysis and interpretation of data, and assisted in the preparation of the manuscript. All authors critically reviewed and approved the final manuscript.

### Conflict of Interest Statement

The authors declare that the research was conducted in the absence of any commercial or financial relationships that could be construed as a potential conflict of interest.
